# Semiquantitative immunohistochemical (IHC) pixelwise H-score of mitochondrial transcription factor A (TFAM) in gastric adenocarcinoma (GAC): clinicopathological significance and association with p53 and HER2

**DOI:** 10.1186/s12957-025-03998-6

**Published:** 2025-12-15

**Authors:** Liang-Hung Ou, Hui-Ting Lee, Chia-I Lin, Yung-Hui Li, Wan-Chun Li, Chen-Sung Lin, Chiang-Ting Chien

**Affiliations:** 1 School of Life Science, National Taiwan Normal University, No. 88, Sec. 4, Ting-Zhou Rd., Wen-Shan Dist., Taipei 116, Taiwan; 2 Division of Allergy, Immunology & Rheumatology, Department of Internal Medicine, Mackay Memorial Hospital, Taipei, 104 Taiwan; 3 School of Medicine,College of Medicine, MacKay Medical University, New Taipei City, 252 Taiwan; 4 General Education Center, National Taipei University of Nursing and Health Sciences, Taipei, 112 Taiwan; 5 Division of Anatomy and Pathology, Taipei Hospital, Ministry of Health and Welfare, New Taipei City, 242 Taiwan; 6 Hon Hai Research Institute, Taipei, 114 Taiwan; 7 Institute of Oral Biology, College of Dentistry, National Yang Ming Chiao Tung University, Taipei, 112 Taiwan; 8 Department of Dentistry, College of Dentistry, National Yang Ming Chiao Tung University, Taipei, 112 Taiwan; 9 Oral Medicine Innovation Center (OMIC), National Yang Ming Chiao Tung University, Taipei, 112 Taiwan; 10 Department of Stomatology, Taipei Veterans General Hospital, Taipei, 112 Taiwan; 11 Division of Thoracic Surgery, Department of Surgery, Taipei Hospital, Ministry of Health and Welfare, No. 127, Si-Yuan Rd., Xin-Zhuang Dist., New Taipei City, 242 Taiwan; 12 School of Medicine, College of Medicine, National Yang Ming Chiao Tung University, Taipei, 112 Taiwan; 13 Center for General Education, Kainan University, Taoyuan City, 338 Taiwan; 14 Division of General Surgery, Department of Surgery, Taipei Hospital, Ministry of Health and Welfare, New Taipei City, 242 Taiwan

**Keywords:** Mitochondrial transcription factor A (TFAM), HER2, p53, Gastric adenocarcinoma (GAC), Warburg effect

## Abstract

**Background:**

The roles of p53 and HER2 in gastric adenocarcinoma (GAC) have been extensively studied; nevertheless, the contribution of mitochondrial transcription factor A (TFAM) remains unclear. Concerning TFAM’s pivotal role in mitochondrial biogenesis, this study aimed to evaluate the TFAM expression in GAC and to assess its associations with p53 and HER2 expressions and clinicopathological outcomes.

**Methods:**

We retrospectively analyzed 77 GAC patients who underwent upfront gastrectomy at Taipei Hospital between 2012 and 2021. Their clinicopathological profiles were recorded in detail. Immunohistochemical (IHC) staining for TFAM, p53, and HER2 protein expressions was semiquantified using IHC pixelwise H-score analyzed by ImageJ plugins IHC profiler. Associations between two continuous variables were assessed by Spearman’s correlation coefficient (CC), and trendlines were fitted using SPSS’s curve estimation function. The optimal cutoff for survival discrimination was derived from receiver operating characteristic (ROC) curve analysis by selecting the threshold with the highest Youden index and area under the curve (AUC). Prognostic variables with a Log-rank test *p*-value ≤ 0.1 were entered into a multi-variate Cox proportional hazards regression (Cox regression) model to identify independent ones and their relative hazards ratio (HR).

**Results:**

TFAM IHC pixelwise H-score was significantly associated with advanced T and N status, lymphovascular invasion, perineural invasion and poor differentiation (all’s *p* < 0.05), and was inversely correlated with tumor size (Spearman’s rho CC = -0.402, *p* < 0.001) in a logarithmic distribution (*p* < 0.001). A positive correlation (Spearman’s rho CC = 0.312, *p* = 0.006) in cubic distribution (*p* < 0.001) was observed between p53and TFAM IHC pixelwise H-scores. ROC analysis yielded a TFAM IHC pixelwise H-score cutoff of 43.0 (AUC = 0.650, 95%CI = 0.515–0.785, *p* = 0.047; sensitivity = 0.490, specificity = 0.810) to dichotomize high and low groups. In multi-variate Cox regression, low TFAM IHC pixelwise H-score (HR = 2.332, 95%CI = 1.136–4.787, *p* = 0.021), M1 status (HR = 3.582, 95%CI = 1.608–7.979, *p* = 0.002), and perineural invasion (HR = 4.506, 95%CI = 1.541–13.177, *p* = 0.006) were identified as independent variables to poor prognosis with elevated HRs. Among patients with high TFAM expression, higher HER2 IHC pixelwise H-score was associated with elevated hazard (HR = 1.010, 95%CI = 1.002–1.019, *p* = 0.020, Cox regression, uni-variate). Among M1 patients, higher p53 IHC pixelwise H-score was related to elevated hazard (HR = 1.029, 95%CI = 1.004–1.056, *p* = 0.025, Cox regression, uni-variate).

**Conclusions:**

ROC and multi-variate Cox regression identified low TFAM expression as an independent poor prognostic variable for operable GAC patients, implying its potential as a quantitative prognostic biomarker. The observed associations with p53 and HER2 are hypothesis-generating and they require further validation to clarify TFAM’s role in Warburg effect and GAC progression.

**Supplementary Information:**

The online version contains supplementary material available at 10.1186/s12957-025-03998-6.

## Introduction

Despite the significant advancements in the modern medicine and public health care, gastric adenocarcinoma (GAC) remains one of the top 10 leading causes of cancer-related deaths worldwide [1]. Extensive research by clinical and surgical oncologists has focused on the molecular mechanisms underlying GAC pathogenesis, particularly the overexpression/or accumulation of protein p53, encoded by the tumor suppressor gene *TP53* [2], the amplification of the human epidermal growth factor receptor 2 (HER2), encoded by the oncogene *ERBB2* [3], or their interplay [4]. However, comparatively little attention has been paid to the potential role of mitochondrial alteration in the initiation and progression of GAC [5].

Dysfunction of cancer cell respiration was first described by Dr. Otto Warburg in the early 20th century, and he discovered that cancer cells exhibit a rapid glucose uptake and an enhanced glycolysis, even in the presence of an ample oxygen supply, a phenomenon known as aerobic glycolysis [6]. This shifting in glucose metabolism, marked by reduced cellular respiration and enhanced glycolysis for ATP production, is known as the Warburg effect [7]. Warburg speculated that defective cellular respiration, now identified as mitochondrial respiratory dysfunction, is a key factor in the carcinogenesis and progression of human cancers [6].

Mitochondrial respiration, the primary process for cellular ATP production, is mainly driven by respiratory enzyme complexes I to V, which comprise 92 protein subunits. Of these, 13 are encoded by mitochondrial DNA (mtDNA), while the remaining 79 are encoded by nuclear DNA (nDNA) [8]. The efficiency of mitochondrial respiration is closely linked to the quantity of mtDNA, as well as the processes of mtDNA transcription and translation [8, 9]. Several proteins are involved in the replication and transcription of mtDNA, among which mitochondrial transcription factor A (TFAM) is an unique one due to its role in both replication and transcription [8, 10]. TFAM is essential for maintaining mitochondrial function [8, 11]. Alterations in TFAM expression may serve as a potential marker to reflect Warburg effect in cancer [12].

Besides its well-known role as a tumor suppressor, p53 could regulate mitochondrial biogenesis by activating its target gene, the peroxisome proliferator-activated receptor gamma coactivator 1-alpha (PGC-1α), which is a key regulator of mitochondrial energy metabolism [13, 14]. PGC-1α could orchestrate the expression of downstream nuclear respiratory factors 1 and 2 (NRF_1/2_) that subsequently regulate TFAM expression, ultimately influencing mitochondrial biogenesis [15].

The oncological effects of HER2 expression, particularly its downstream impact on the PI3K/Akt pathway (PI3K, Phosphoinositide 3-kinase; Akt, Protein kinase B), have been extensively studied in breast cancer [16, 17]. Recently, the HER2/PI3K/Akt pathway and HER2-targeted therapies have gained increasing attention in GAC [18]. Of note, Akt could promote cellular glucose uptake and is regarded as a key role in driving the Warburg effect in human cancers [19].

In this retrospective analysis, we investigated the clinicopathological significance of TFAM expression in GAC and its association with p53 and HER2 expression by applying a semiquantitative immunohistochemical (IHC) pixelwise H-score. Additionally, we aimed to elucidate the potential molecular interplay among TFAM, HER2 and p53 underlying GAC pathogenesis and prognosis from the viewpoint of TFAM-related Warburg effect.

## Materials and methods

### Patient information

**Fig. 1:**
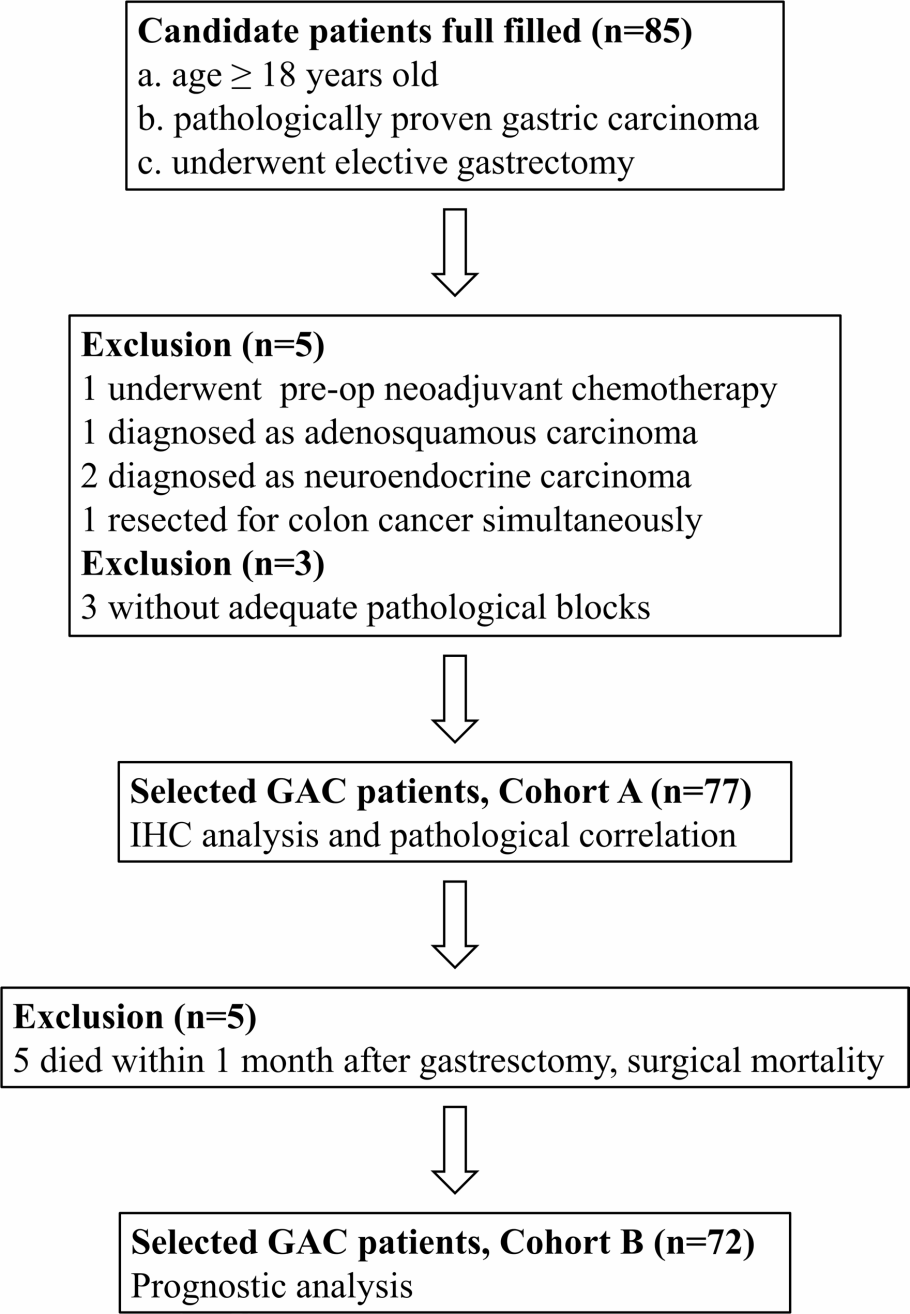
Flowchart illustrates the inclusion and exclusion criteria for recruiting GAC patients. A total of 77 cases were included in Cohort A for IHC analysis and pathological correlation, and 72 cases were included in Cohort B for prognostic evaluation. GAC, gastric adenocarcinoma; IHC, immunohistochemical

Candidate patients were selected from the Division of General Surgery, Department of Surgery, Taipei Hospital, Ministry of Health and Welfare, New Taipei City, Taiwan, between January 2012 and June 2021. Inclusion criteria required patients to be over 18 years old, have a pathologically confirmed diagnosis of gastric carcinoma, and have initially undergone elective gastrectomy (*n* = 85). Exclusion criteria included those who received preoperative neoadjuvant chemotherapy (*n* = 1), had a diagnosis of adenosquamous carcinoma (*n* = 1) or neuroendocrine carcinoma (*n* = 2) other than GAC, underwent synchronous resection of colorectal cancer (*n* = 1), or had inadequately preserved pathological blocks (*n* = 3). A total of 77 consecutive GAC patients were included in Cohort A (Fig. 1), and their clinicopathological profiles were recorded in detail. Pathological tissue samples from Cohort A were subjected to IHC staining for further pathological and clinical correlation. Among these, 5 GAC patients (5/77, 6.5% of Cohort A cases, or 5/85, 5.9% of overall gastrectomy cases) were excluded due to surgical mortality, as they died within one month after surgery. Consequently, 72 GAC patients were recruited into Cohort B (Fig. 1) for prognostic analysis. This retrospective study and the waiver of the participants’ consent was approved by the Institutional Review Board of Taipei Hospital, Ministry of Health and Welfare, New Taipei City, Taiwan (Approval Nos. TH-IRB-0018-0022, TH-IRB-0022-0010, and TH-IRB-0024-002), as well as the Institutional Review Board of Taoyuan Hospital, Ministry of Health and Welfare, Taoyuan City, Taiwan (Approval No. TYGH-114026). All patients were followed up until November 15, 2024.

### Gastrectomy, lymph node dissection and pathological diagnosis

In Cohort A, surgeries included either subtotal (*n* = 56, 72.7%) or total (*n* = 21, 27.3%) gastrectomy, with the creation of gastroesophageal, Billroth-I gastroduodenal, or Billroth-II/Roux-en-Y gastrojejunal/esophagojejunal anastomoses.

Lymph node dissection was performed along the left gastric artery, common hepatic artery, celiac artery, splenic artery, and hepatoduodenal ligament, similar to protocols about D1, D1^+^, or D2 dissections in the literatures [20, 21].

Their pathological T-, N-, and M-status, as well as cancer stage, were described according to the AJCC 8th edition. Additionally, the pathological conditions of lymphovascular invasion, perineural invasion, and cell differentiation were assessed [22].

### IHC staining and analysis

Pathological slides were reviewed by an experienced pathologist, Dr. C.-I. Lin, who identified representative tumor foci of GAC. Thin sections (~ 3 μm) were prepared from the corresponding formalin-fixed, paraffin-embedded tissue blocks and subjected to IHC staining.

IHC staining was carried out through a standardized protocol on the Leica BOND MAX automated staining platform (BOND BIOTECH, INC, Taiwan; https://bondbiotech.com.tw/). First, the tissue sections were de-paraffinized and rehydrated through a series of ethanol washes. Antigen retrieval was carried out by using an EDTA solution (Leica ER2) at pH 9.0, heated to 95 °C for 30 min. Endogenous peroxidase activity was then blocked with a 3–4% hydrogen peroxide solution (Leica) for 5 min. For primary staining, sections were incubated with the following antibodies: TFAM (polyclonal, Sigma, cat. no. HPA040648) at a 1:50 dilution, p53 (clone DO7, Leica, cat. no. NCL-L-p53-DO7) at a 1:50 dilution, and HER2 (clone SP3, Zytomed, cat. no. RBK026) at a 1:200 dilution, respectively, for 55 min at room temperature. Secondary staining was performed by incubating sections with secondary antibodies conjugated to peroxidase (Leica) for 20 min at room temperature. Finally, the stained proteins were detected using 3,3’-Diaminobenzidine (DAB) chromogen for 5 min, and the sections were counterstained with hematoxylin. Representative positive IHC staining for TFAM, HER2, and p53, asl well as the internal negative control, are shown in Supplemental Fig. 1.

The relative protein expression levels were determined semiquantitatively using the IHC pixelwise H-score, as described and modified from the published literature [23–25]. The IHC pixelwise H-score was calculated using the following formula:

IHC pixelwise H-score = (3 × percentage of high positive cells) + (2 × percentage of positive cells) + (1 × percentage of low positive cells) + (0 × percentage of negative cells), with a range of 0 ≤ IHC pixelwise H-score ≤ 300.

### Whole slide image (WSI), region of interest (ROI) and IHC pixelwise H-score

**Fig. 2:**
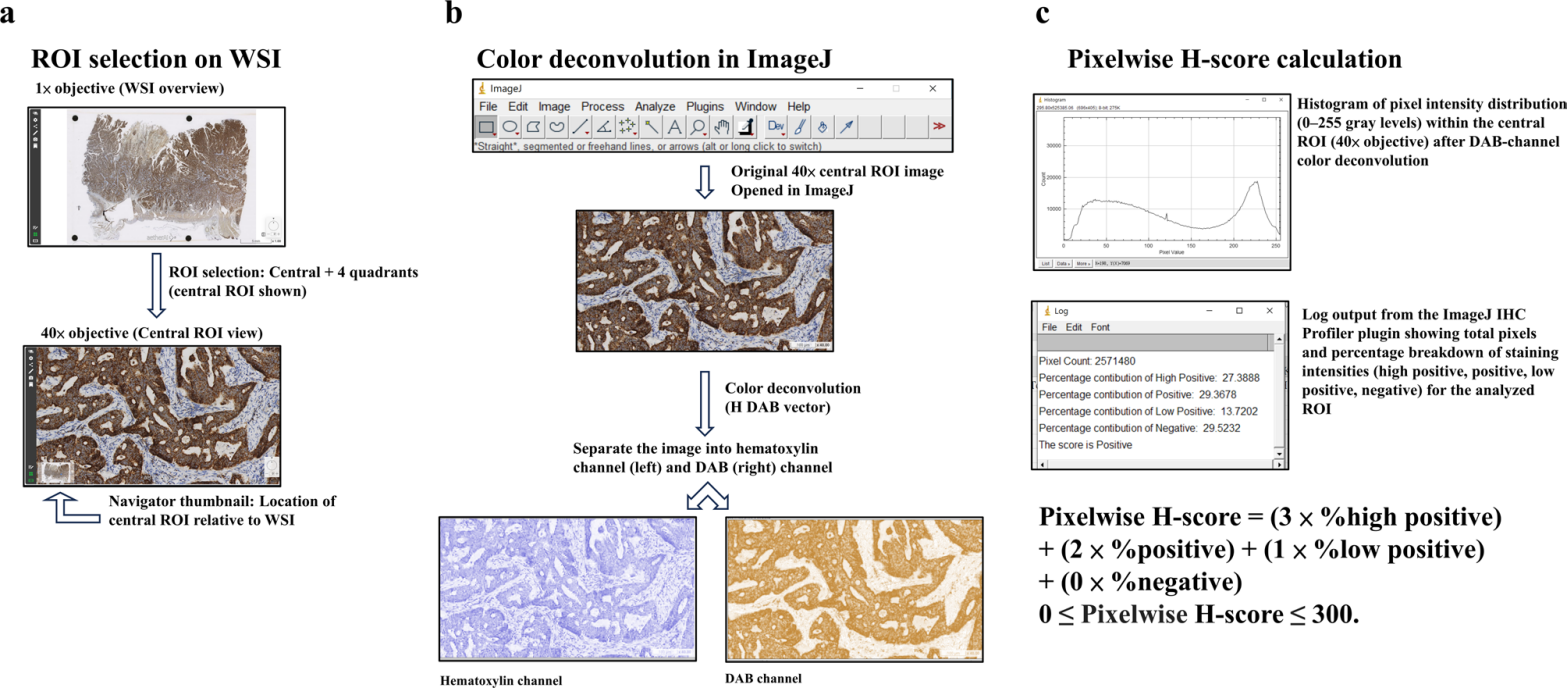
Workflow for semiquantitative IHC pixelwise H-score analysis. Example of TFAM IHC pixelwise H-score in GAC case 68 is demonstrated. (a) ROI selection on WSI. A low-power overview (1× objective) of a TFAM-stained GAC WSI is shown at top panel. Five ROIs were sampled using a central + four-quadrant scheme. The central ROI is displayed at 40× objective on the bottom panel. The navigator thumbnail (bottom left of the 40× view) indicates the ROI’s location within the full WSI. (b) Color deconvolution in ImageJ. The 40× central-ROI image (middle panel) was opened in ImageJ (top panel) and processed via IHC Profiler plugins, yielding separate hematoxylin (left) and DAB (right) channel images (bottom panel) for quantification. (c) Pixelwise H-score calculation. The DAB-channel ROI was analyzed with the IHC Profiler plugin, which classifies pixels as high positive, positive, low positive, or negative. A representative pixel-intensity histogram (0–255 gray levels) shows the distribution within the ROI (top panel), and the plugin’s log output reports the percentage of pixels in each category (middle panel). The overall pixelwise H-score is calculated as (low panel) : Pixelwise H-score = (3 × %high positive) + (2 × %positive) + (1 × %low positive) + (0 × %negative); 0 ≤ Pixelwise H-score ≤ 300. The TFAM IHC pixelwise H-score of the central ROI was then calculated as follows: (27.3888 × 3) + (29.3678 × 2) + (13.7202 × 1) + (29.5232 × 0) = 154.6. GAC, gastric adenocarcinoma; IHC, immunohistochemical; ROI, region of interest; TFAM, mitochondrial transcription factor A; WSI, whole slide image; DAB, 3,3′-diaminobenzidine

The light microscopic fields of IHC-stained slides were digitalized into WSIs (AetherAI Co., Ltd, Taipei, Taiwan; https://www.aetherai.com/). Each WSI was magnified to 400x (a combination of a 40x objective lens and a 10x ocular lens) to select representative areas within GAC nest as ROI (Fig. 2, a). ROIs were manually identified on WSIs by an experienced pathologist (Dr. C-I Lin) in multidisciplinary consensus with a general surgeon (Dr. L-H Ou), an allergy/immunology/rheumatology physician (Dr. H-T Lee), and a thoracic surgeon (Dr. C-S Lin), rather than by independent blinded review, to promote consistency and reduce selection bias. ROIs were chosen to include predominantly viable tumor nests and to exclude areas with obvious necrosis or dense lymphocytic infiltration. For sufficiently large tumors, a “central plus four quadrants” sampling strategy was applied, and up to 5 ROIs were selected to capture intra-tumoral heterogeneity; nevertheless, smaller lesions (e.g., T1) permitted fewer ROIs. The number of ROIs per case therefore ranged from 1 to 5. The selected ROIs were exported as PNG images for subsequent calculation of the IHC pixelwise H-score. For the 77 HER2 slides, the number of selected ROIs per WSI case was as follows: 1 ROI for 18 cases, 2 ROIs for 4 cases, 3 ROIs for 2 cases, 4 ROIs for 1 case, and 5 ROIs for 52 cases (Medium, 5 ROIs). For the 77 TFAM slides, the selection included: 1 ROI for 13 cases, 2 ROIs for 3 cases, 3 ROIs for 2 cases, and 5 ROIs for 59 cases (Medium, 5 ROIs). For the 77 p53 slides, the selection included: 1 ROI for 14 cases, 2 ROIs for 2 cases, 3 ROIs for 3 cases, and 5 ROIs for 58 cases (Medium, 5 ROIs).

With the assistance of Dr. Yung-Hui Li, an expert in image processing, the captured images were imported into ImageJ and analyzed using the IHC Profiler plugin, as described in the literature [23, 25]. The percentages of high positive, positive, low positive, and negative expression for the target protein in each image (ROI) were then determined (Fig. 2, b & c) to calculate the IHC pixelwise H-score [23, 25]. The mean of IHC pixelwise H-scores of analyzed ROIs was used to represent the expression level of the target protein for each IHC slide.

### Prognostic variables

Possible prognostic variables, including sex, age, type of gastrectomy, pathological findings, and IHC pixelwise H-scores of TFAM, HER2, and p53, were analyzed to identify independent ones.

### Statistical analysis

Overall survival was calculated from the date of surgery to either death or the last follow-up on November 15, 2024. Survival curves were generated using the Kaplan–Meier method, and differences between groups were compared with the Log-rank test. A uni-variate Cox proportional hazards regression model (Cox regression model) was used to calculate relative hazard ratios (HRs) for each variable. Variables with a *p*-value ≤ 0.1 in the Log-rank test were included in a multi-variate Cox regression model to identify independent prognostic variables and determine their relative HRs. Categorical variables, analyzed across two or more groups, were assessed using the χ² test or Fisher’s exact test, as appropriate. The normality of continuous variables was evaluated with the Kolmogorov–Smirnov test (for sample sizes > 50), where a *p*-value < 0.05 indicated a non-normal distribution [26]. Continuous variables were presented as either mean ± standard deviation (STD), median (25th percentile, 75th percentile; [25P, 75P]), or mean (95% confidence interval [CI]), as appropriate. Comparisons of continuous variables across groups were performed using *t*-test, Mann–Whitney *U* test, analysis of variance (ANOVA), or Kruskal–Wallis *H* test, depending on data distribution. Correlations between two continuous variables were assessed using parametric Pearson’s or non-parametric Spearman’s rho correlation coefficients (CC), based on data characteristics. Curve fit analysis was performed using the Curve Estimation function in SPSS. A variety of regression models were tested, including linear, quadratic, cubic, logarithmic, inverse, power, exponential, compound, growth, logistic, and S models. The best-fit model was selected based on the highest *R²* value, largest F statistic and lowest *p*-value. The selected model was subsequently applied to generate scatter plots with corresponding fitted trendlines for graphical visualization. The optimal cutoff value for the IHC pixelwise H-score of the target protein (e.g., TFAM) to differentiate survival status (alive vs. deceased) in GAC patients was determined using the area under the curve (AUC) and the Youden index from a receiver operating characteristic (ROC) curve [27]. Statistical significance was set at *p* < 0.05. All statistical analyses were conducted using SPSS software (SPSS Statistics for Windows, Version 17.0, SPSS Inc., Chicago, IL, USA).

## Results

### Demographic data of Cohort A GAC patients (n = 77)

Cohort A consisted of 77 GAC patients with a mean age of 68.0 years (Female/Male = 26/51) (Table 1). Among them, 21 patients (27.3%) underwent total gastrectomy, while 56 (72.7%) received subtotal gastrectomy. Regarding their pathological findings for T-status, 14 patients (18.2%) were diagnosed as T1, 6 (7.8%) as T2, 39 (50.6%) as T3, and 18 (23.4%) as T4. For N status, 24 patients (31.2%) were N0, 5 (6.5%) were N1, 13 (16.9%) were N2, and 35 (45.5%) were N3. In terms of M status, 63 patients (81.8%) were M0, while 14 (18.2%) were M1. Forty-nine patients (63.6%) had a lymphovascular invasion, and 50 (64.9%) had a perineural invasion. Regarding GAC cell differentiation, 10 patients (13.0%) had a well-differentiated pattern, 29 (37.7%) had a moderately differentiated pattern, and 38 (49.4%) had a poorly differentiated pattern (Table 1). Their mean maximal tumor diameter was 5.1 cm. The distribution of IHC pixelwise H-scores for TFAM, HER2, and p53 was non-normal. The means and medians were as follows: TFAM (mean = 54.8, median = 51.8), HER2 (mean = 20.2, median = 2.6), and p53 (mean = 31.6, median = 15.1) (Table 1).

**Table 1:** Demographic data of the Cohort A GAC patients (*n* = 77)

	Case number (%), Mean ± STD/Median (25P, 75P)
Gender	
Female/Male	26 (33.8)/51 (66.2)
Age (years)	68.0 ± 12.7/69.0 (60.5, 77.0)
Type of gastrectomy	
Total/Subtotal	21 (27.3)/56 (72.7)
Pathological findings	
T-status (AJCC 8th)	
T1/T2/T3/T4	14 (18.2)/6 (7.8)/39 (50.6)/18 (23.4)
N-status (AJCC 8th)	
N0/N1/N2/N3	24 (31.2)/5 (6.5)/13 (16.9)/35 (45.5)
M-status (AJCC 8th)	
M0/M1	63 (81.8)/14 (18.2)
Lymphovascular invasion	
No/Yes	28 (36.4)/49 (63.6)
Perineural invasion	
No/Yes	27 (35.1)/50 (64.9)
Cell differentiation	
Well/Moderate/Poor	10 (13.0)/29 (37.7)/38 (49.4)
Maximal tumor diameter (cm)	5.1 ± 3.1/5.0 (2.4, 7.2)
IHC pixelwise H-score	
TFAM*	54.8 ± 37.3/51.8 (29.0, 68.2)
HER2*	20.2 ± 39.8/2.6 (1.1, 17.9)
p53*	31.6 ± 33.4/15.1 (7.5, 50.4)

AJCC, American Joint Committee on Cancer; GAC, gastric adenocarcinoma; HER2, human epidermal growth factor receptor 2; IHC, immunohistochemical; 25P, 25th percentile; 75P, 75th percentile; STD, standard deviation; TFAM, mitochondrial transcription factor A

*Non-normal distribution, *p* < 0.001 (Kolmogorov-Smirnov test)

### Distribution of TFAM, HER2, and p53 IHC pixelwise H-scores in Cohort A GAC patients based on pathological findings (n = 77)

Regarding the distribution of TFAM IHC pixelwise H-scores, we observed a trend of decreasing scores with the progression of T status (*p* = 0.003, Kruskal–Wallis *H* test), N status (*p* = 0.003, Kruskal–Wallis *H* test), lymphovascular invasion (*p* = 0.009, Mann–Whitney *U* test), perineural invasion (*p* < 0.001, Mann–Whitney *U* test), and poor cellular differentiation (*p* < 0.001, Kruskal–Wallis *H* test) (Table 2, upper part, left side). No similar decrease was observed for the HER2 IHC pixelwise H-score, except for poor cellular differentiation (*p* = 0.001, Kruskal–Wallis *H* test) (Table 2, middle part, left side); nor for the p53 IHC pixelwise H-score, except for poor cellular differentiation (*p* = 0.013, Kruskal–Wallis *H* test) (Table 2, lower part, left side). Of note, lower IHC pixelwise H-scores for TFAM, HER2, and p53 were all associated with poor cellular differentiation in GAC.

**Table 2:** Distribution of TFAM, HER2, and p53 IHC pixelwise H-scores in Cohort A GAC patients based on pathological findings (*n* = 77)

Pathological findings	TFAM IHC pixelwise H-score (Medium = 51.8)				
(Case number, %)	Mean ± STD/Median (25P, 75P)	*p*-value^a^	≤ 51.8 (*n* = 39)	> 51.8 (*n* = 38)	*p*-value^b^
T-status (AJCC 8th)		0.003			0.011
T1 (*n* = 14, 100.0)	81.6 *±* 40.7/64.9 (55.5, 90.6)		2 (14.3)	12 (85.7)	
T2 (*n* = 6, 100.0)	75.3 ± 38.4/76.6 (40.0, 113.9)		2 (33.3)	4 (66.7)	
T3 (*n* = 39, 100.0)	45.4 ± 33.7/42.2 (21.8, 58.0)		25 (64.1)	14 (35.9)	
T4 (*n* = 18, 100.0)	47.5 ± 31.3/40.7 (19.8, 72.0)		10 (55.6)	8 (44.4)	
N-status (AJCC 8th)		0.003			0.004
N0 (*n* = 24, 100.0)	79.1 ± 42.6/62.4 (53.8, 111.5)		5 (20.8)	19 (79.2)	
N1 (*n* = 5, 100.0)	52.8 ± 39.4/51.8 (17.5, 88.7)		3 (60.0)	2 (40.0)	
N2 (*n* = 13, 100.0)	47.7 ± 31.4/50.9 (15.8, 70.7)		7 (53.8)	6 (46.2)	
N3 (*n* = 35, 100.0)	41.1 ± 26.9/34.4 (21.2, 61.2)		24 (68.6)	11 (31.4)	
M-status (AJCC 8th)		0.383			0.591
M0 (*n* = 63, 100.0)	57.0 ± 38.6/52.7 (31.9, 71.2)		31 (49.2)	32 (50.8)	
M1 (*n* = 14, 100.0)	44.8 ± 30.2/35.0 (20.4, 66.7)		8 (57.1)	6 (42.9)	
Lymphovascular invasion		0.009			0.014
No (*n* = 28, 100.0)	71.5 ± 44.0/55.9 (47.4, 87.3)		9 (32.1)	19 (67.9)	
Yes (*n* = 49, 100.0)	45.3 ± 29.3/40.3 (21.0, 64.2)		30 (61.2)	19 (38.8)	
Perineural invasion		< 0.001			< 0.001
No (*n* = 27, 100.0)	77.9 ± 42.3/64.7 (52.7, 108.8)		6 (22.2)	21 (77.8)	
Yes (*n* = 50, 100.0)	42.4 ± 27.5/41.3 (20.3, 58.3)		33 (66.0)	17 (34.0)	
Differentiation		< 0.001			< 0.001
Well (*n* = 10, 100.0)	88.1 ± 42.8/68.4 (53.9, 119.4)		1 (10.0)	9 (90.0)	
Moderate (*n* = 29,100.0)	67.9 ± 38.7/63.6 (45.9, 85.9)		8 (27.6)	21 (72.4)	
Poor (*n* = 38, 100.0)	36.0 ± 21.9/33.6 (18.8, 51.1)		30 (78.9)	8 (21.1)	

Pathological findings	HER2 IHC pixelwise H-score (Medium = 2.6)				
(Case number, %)	Mean ± STD/Median (25P, 75P)	*p*-value^a^	≤ 2.6 (*n* = 39)	> 2.6 (*n* = 38)	*p*-value^b^
T-status (AJCC 8th)		0.897			0.775
T1 (*n* = 14, 100.0)	11.3 ± 15.3/3.3 (0.7, 19.6)		7 (50.0)	7 (50.0)	
T2 (*n* = 6, 100.0)	7.5 ± 10.6/1.7 (0.8, 16.8)		4 (66.7)	2 (33.3)	
T3 (*n* = 39, 100.0)	20.3 ± 39.6/4.3 (1.4, 15.3)		18 (46.2)	21 (53.8)	
T4 (*n* = 18, 100.0)	31.0 ± 55.6/1.7 (0.8, 41.9)		10 (55.6)	8 (44.4)	
N-status (AJCC 8th)		0.495			0.181
N0 (*n* = 24, 100.0)	9.3 ± 13.3/2.3 (1.0, 12.5)		14 (58.3)	10 (41.7)	
N1 (*n* = 5, 100.0)	14.4 ± 19.7/0.8 (0.6, 35.1)		3 (60.0)	2 (40.0)	
N2 (*n* = 13, 100.0)	25.9 ± 42.6/8.3 (2.9, 24.1)		3 (23.1)	10 (76.9)	
N3 (*n* = 35, 100.0)	26.3 ± 50.9/2.0 (1.2, 22.0)		19 (54.3)	16 (45.7)	
M-status (AJCC 8th)		0.968			0.591
M0 (*n* = 63, 100.0)	19.9 ± 38.5/3.8 (1.1, 18.6)		31 (49.2)	32 (50.8)	
M1 (*n* = 14, 100.0)	21.4 ± 46.6/2.2 (1.5, 13.6)		8 (57.1)	6 (42.9)	
Lymphovascular invasion		0.403			0.698
No (*n* = 28, 100.0)	9.4 ± 13.0/2.4 (0.9, 12.5)		15 (53.6)	13 (46.4)	
Yes (*n* = 49, 100.0)	26.3 ± 48.0/3.8 (1.2, 22.2)		24 (49.0)	25 (51.0)	
Perineural invasion		0.831			0.201
No (*n* = 27, 100.0)	11.7 ± 14.2/7.2 (0.8, 17.2)		11 (40.7)	16 (59.3)	
Yes (*n* = 50, 100.0)	24.7 ± 47.8/2.1 (1.2, 19.5)		28 (56.0)	22 (44.0)	
Differentiation		0.001			0.008
Well (*n* = 10, 100.0)	12.8 ± 14.8/7.9 (1.2, 21.3)		4 (40.0)	6 (60.0)	
Moderate (*n* = 29,100.0)	40.4 ± 57.1/11.0 (1.8, 54.5)		9 (31.0)	20 (69.0)	
Poor (*n* = 38, 100.0)	6.7 ± 14.4/1.6 (0.7, 4.2)		26 (68.4)	12 (31.6)	

Pathological findings	p53 IHC pixelwise H-score (Medium = 15.1)				
(Case number, %)	Mean ± STD/Median (25P, 75P)	*p*-value^a^	≤ 15.1 (*n* = 39)	> 15.1 (*n* = 38)	*p*-value^b^
T-status (AJCC 8th)		0.316			0.756
T1 (*n* = 14, 100.0)	31.0 ± 39.0/10.5 (5.8, 57.1)		8 (57.1)	6 (42.9)	
T2 (*n* = 6, 100.0)	67.3 ± 57.2/59.5 (9.4, 127.5)		2 (33.3)	4 (66.7)	
T3 (*n* = 39, 100.0)	25.2 ± 21.0/15.3 (8.2, 38.3)		19 (48.7)	20 (51.3)	
T4 (*n* = 18, 100.0)	34.2 ± 36.4/14.4 (7.4, 61.6)		10 (55.6)	8 (44.4)	
N-status (AJCC 8th)		0.370			0.124
N0 (*n* = 24, 100.0)	36.7 ± 41.5/16.1 (7.3, 48.9)		11 (45.8)	13 (54.2)	
N1 (*n* = 5, 100.0)	62.1 ± 48.6/69.4 (14.0, 106.7)		1 (20.0)	4 (80.0)	
N2 (*n* = 13, 100.0)	17.8 ± 19.4/11.3 (8.4, 15.2)		10 (76.9)	3 (23.1)	
N3 (*n* = 35, 100.0)	29.0 ± 26.1/20.5 (7.5, 51.8)		17 (48.6)	18 (51.4)	
M-status (AJCC 8th)		0.827			0.957
M0 (*n* = 63, 100.0)	32.7 ± 34.6/15.1 (7.2, 51.8)		32 (50.8)	31 (49.2)	
M1 (*n* = 14, 100.0)	26.7 ± 27.9/16.5 (8.0, 34.4)		7 (50.0)	7 (50.0)	
Lymphovascular invasion		0.394			0.301
No (*n* = 28, 100.0)	38.1 ± 42.2/19.3 (8.6, 48.9)		12 (42.9)	16 (57.1)	
Yes (*n* = 49, 100.0)	27.9 ± 26.9/12.5 (7.4, 51.5)		27 (55.1)	22 (44.9)	
Perineural invasion		0.597			0.877
No (*n* = 27, 100.0)	36.7 ± 40.2/14.9 (8.2, 63.0)		14 (51.9)	13 (48.1)	
Yes (*n* = 50, 100.0)	28.9 ± 29.10/15.2 (7.4, 42.0)		25 (50.0)	25 (50.0)	
Differentiation		0.013			0.407
Well (*n* = 10, 100.0)	45.1 ± 50.5/15.0 (10.7, 93.1)		5 (50.0)	5 (50.0)	
Moderate (*n* = 29,100.0)	38.1 ± 33.1/28.6 (10.9, 61.6)		12 (41.4)	17 (58.6)	
Poor (*n* = 38, 100.0)	23.1 ± 26.2/8.4 (6.3, 37.6)		22 (57.9)	16 (42.1)	

AJCC, American Joint Committee on Cancer; GAC, gastric adenocarcinoma; HER2, human epidermal growth factor receptor 2; IHC, immunohistochemical; 25P, 25 percentile; 75P, 75 percentile; STD, standard deviation; TFAM, mitochondrial transcription factor A

^a^Kruskal-Wallis *H* test or Mann-Whitney *U* Test; ^b^χ^2^ test

Using the median as a cutoff to classify high and low TFAM IHC pixelwise H-scores, we found that a low TFAM IHC pixelwise score (≤ 51.8) was significantly associated with advanced T status (*p* = 0.011, χ² test), advanced N status (*p* = 0.004, χ² test), lymphovascular invasion (*p* = 0.014, χ² test), perineural invasion (*p* < 0.001, χ² test), and poor cellular differentiation (*p* < 0.001, χ² test) (Table 2, upper part, right side). However, similar associations were not found for a low HER2 IHC pixelwise H-score, except for poor cellular differentiation (*p* = 0.008, χ² test) (Table 2, middle part, right side). No associations were observed for a low p53 IHC pixelwise H-score (Table 2, lower part, right side).

### Associations between maximal tumor diameter and IHC pixelwise H-scores of TFAM, HER2, and p53, and between TFAM IHC pixelwise H-scores and those of HER2 and p53 in Cohort A GAC patients (n = 77)

**Fig. 3:**
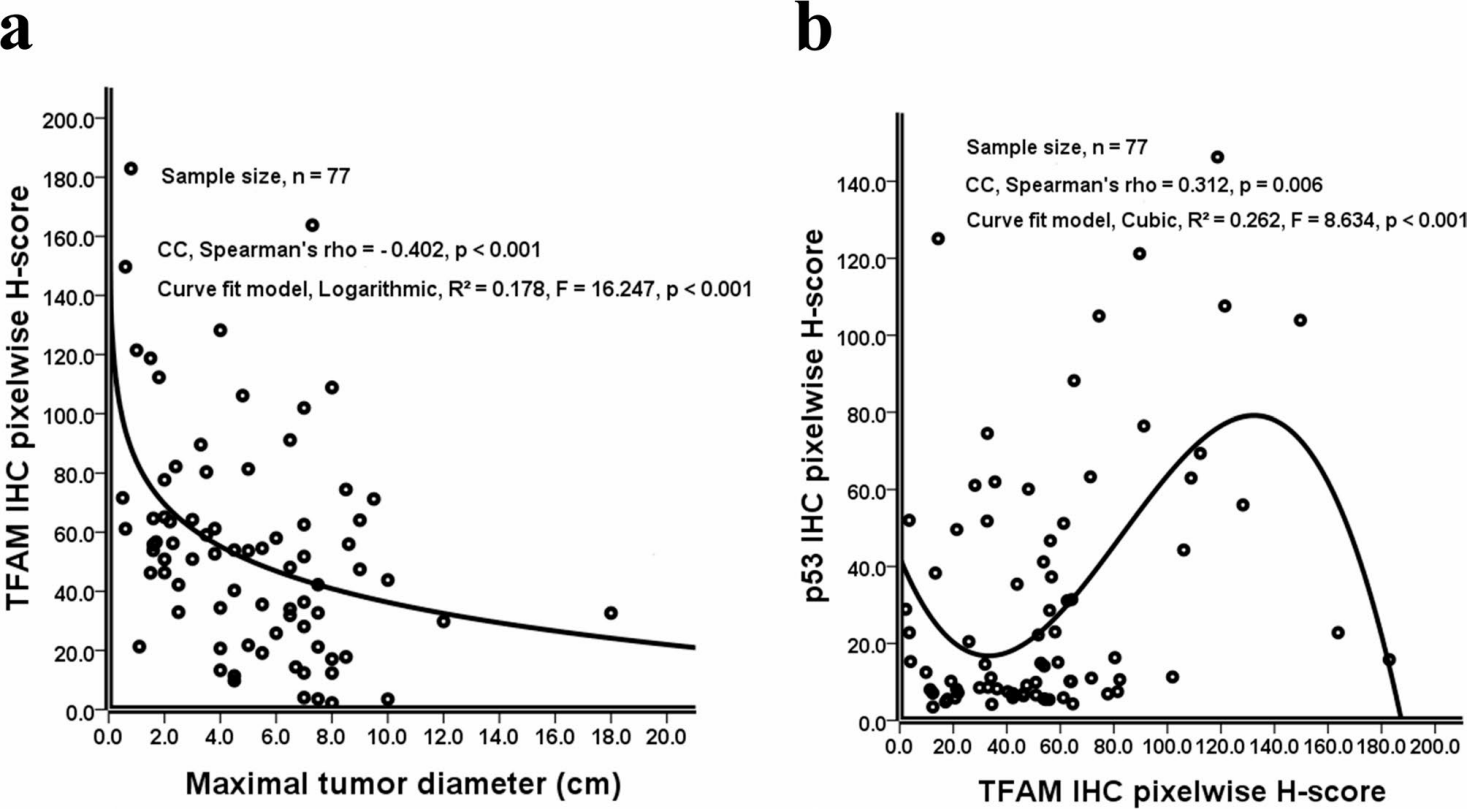
(a) In Cohort A of 77 GAC patients, a significant negative correlation was observed between TFAM IHC pixelwise H-scores (Y-axis) and maximal tumor diameters (X-axis, cm), based on non-parametric Spearman’s rank correlation (CC, Spearman’s Rho=−0.402, *p* < 0.001). A regression analysis evaluating multiple curve fitting models identified the logarithmic model as the best fit one (*R²*=0.178, *p* < 0.001). The logarithmic trendline is displayed as a solid black line. (b) In the same cohort, a significant positive correlation was observed between p53 IHC pixelwise H-scores (Y-axis) and TFAM IHC pixelwise H-scores (X-axis), based on non-parametric Spearman’s rank correlation (CC, Spearman’s Rho = 0.312, *p* = 0.006). A regression analysis evaluating multiple curve fitting models identified the cubic model as the best fit one (*R²*=0.262, *p* < 0.001). The cubic trendline is displayed as a solid black line. GAC, gastric adenocarcinoma; CC, correlation coefficient; IHC, immunohistochemical; TFAM, mitochondrial transcription factor A

As shown in Table 3; Fig. 3 (a), a lower TFAM IHC pixelwise H-score (Y-axis) was correlated with a larger maximal tumor diameter (X-axis, cm, Non-parametric, Spearman’s rho CC = −0.402, *p* < 0.001). This negative correlation followed a logarithmic distribution, which provided the best fit for the data (*R*^*2*^ = 0.178, F = 16.247 and *p* < 0.001). However, no such correlation was observed between HER2 (*p* = 0.513) or p53 (*p* = 0.525) IHC pixelwise H-scores and maximal tumor diameter (Table 3).

**Table 3:** Associations between maximal tumor diameter and IHC pixelwise H-scores of TFAM, HER2, and p53, and between TFAM IHC pixelwise H-scores and those of HER2 and p53 in Cohort A GAC patients (*n* = 77)

	Correlation		Curve fit model			
	CC (Spearman’s rho)	*p*-value		*R* ^2^	F-value	*p*-value
Association to maximal tumor diameter						
TFAM IHC pixelwise H-score	−0.402	< 0.001	Logarithmic*	0.178	16.247	< 0.001*
HER2 IHC pixelwise H-score	−0.076	0.513				
p53 IHC pixelwise H-score	0.074	0.525				
Association to TFAM IHC pixelwise H-score						
HER2 IHC pixelwise H-score	0.110	0.357				
p53 IHC pixelwise H-score	0.312	0.006	Cubic**	0.262	8.634	< 0.001**

CC, correlation coefficient; GAC, gastric adenocarcinoma; HER2, human epidermal growth factor receptor 2; IHC, immunohistochemical; TFAM, mitochondrial transcription factor A

*Through several curve estimation models set in SPSS, the logarithmic model had the highest *R*^*2*^ of 0.178, largest F of 16.247 and a lowest *p*-value of < 0.001

**Through several curve estimation models set in SPSS, the cubic model had the highest *R*^*2*^ of 0.262, largest F of 8.634 and a lowest *p*-value of < 0.001

As shown in Table 3; Fig. 3 (b), a higher p53 IHC pixelwise H-score (Y-axis) was associated with a higher TFAM IHC pixelwise H-score (X-axis, Non-parametric, Spearman’s rho CC = 0.312, *p* = 0.006). This positive correlation followed a cubic distribution, which was identified as the optimal curve fit (*R*^*2*^ = 0.262, F = 8.634 and *p* < 0.001). However, no significant association was found between the HER2 IHC pixelwise H-score and the TFAM IHC pixelwise H-score (*p* = 0.357, Table 3).

### Impact of TFAM, HER2, and p53 IHC pixelwise H-scores on survival HRs and differences in Cohort B GAC patients (n = 72)

As shown in Table 4, in the continuous model, a higher TFAM IHC pixelwise H-score tended to associate with a reduced hazard of death (HR = 0.990; 95%CI = 0.980-1.000; *p* = 0.057, Cox regression, uni-variate). In contrast, HER2 (*p* = 0.236, Cox regression, uni-variate) and p53 (*p* = 0.785, Cox regression, uni-variate) IHC pixelwise H-scores were not significantly associated with survival hazards.

**Table 4:** Impact of TFAM, HER2, and p53 IHC pixelwise H-Scores on survival HRs in Cohort B GAC patients (*n* = 72)

IHC pixelwise H-score	Cox regression model	
	HRs (95%CI)	*p*-value (uni-variate)
Continuous model		
TFAM	0.990 (0.980–1.000)	0.057
HER2	1.004 (0.998–1.010)	0.236
p53	0.999 (0.991–1.007)	0.785
Categorical model		
TFAM		0.005
Low (IHC pixelwise H-score ≤ 43.0) (*n* = 29, 40.3%)*	2.223 (1.272–3.884)	
High (IHC pixelwise H-score > 43.0) (*n* = 43, 59.7%)*	1.000	
HER2		0.235
Low (IHC pixelwise H-score ≤ 2.4) (*n* = 36, 50.0%)**	1.399 (0.804–2.436)	
High (IHC pixelwise H-score > 2.4) (*n* = 36, 50.0%)**	1.000	
P53		0.668
Low (IHC pixelwise H-score ≤ 14.8) (*n* = 36, 50.0%)***	0.886 (0.509–1.542)	
High (IHC pixelwise H-score > 14.8) (*n* = 36, 50.0%)***	1.000	

HRs, hazard ratios; GAC, gastric adenocarcinoma; HER2, human epidermal growth factor receptor 2; IHC, immunohistochemical; TFAM, mitochondrial transcription factor A

*Using ROC analysis, various cutoff points for the TFAM IHC pixelwise H-score were evaluated, and 43.0 was selected as the optimal threshold based on the highest Youden index (0.300; sensitivity = 0.490, specificity = 0.810). This cutoff corresponded to the largest AUC (0.650; 95% CI = 0.515–0.785; *p* = 0.047) and was used to dichotomize TFAM IHC pixelwise H-score into low and high groups

**The median HER2 IHC pixelwise H-score was 2.4; this value was used as the cutoff to dichotomize HER2 IHC pixelwise H-score into low and high groups

***The median p53 IHC pixelwise H-score was 14.8; this value was used as the cutoff to dichotomize p53 IHC pixelwise H-score into low and high groups

Using ROC curves, we tested various cutoff points for the TFAM IHC pixelwise H-score and identified 43.0 as the optimal threshold, yielding the highest Youden index (0.300; Sensitivity = 0.490, Specificity = 0.810) with largest AUC (0.650, 95%CI = 0.515–0.785, *p* = 0.047). Patients with a TFAM IHC pixelwise H-score ≤ 43.0 were classified as the low TFAM group (*n* = 29), while those with a score > 43.0 were classified as the high TFAM group (*n* = 43). The low TFAM group exhibited a significantly higher hazard of death (HR = 2.223, 95%CI = 1.272–3.884, *p* = 0.005, Cox regression, uni-variate) compared to that of the high TFAM group (Table 4). Kaplan–Meier survival analysis showed that the low TFAM group had significantly shorter overall survival (mean = 30.5, 95%CI = 14.2–46.8, months) compared with the high TFAM group (mean = 63.2, 95%CI = 45.3–81.2, months, *p* = 0.004, Log-rank) (Supplemental Fig. 2a; Table 5).

**Table 5:** Prognostic and independent variables in Cohort B GAC patients (n=72)

Prognostic variables (case number, %)	Survival difference		Cox regression model	
	Survival (months), Mean (95%CI)	*p*-value (Log-rank)	HRs (95%CI)	*p*-value (multi-variate)
Gender		0.602		
Female (*n* = 26, 36.1)	50.1 (31.1–69.0)			
Male (*n* = 46, 63.9)	46.8 (31.4–62.2)			
Age		0.103		
≤65 (*n* = 31, 43.1)	65.4 (42.0– 88.7)			
>65 (*n* = 41, 56.9)	37.3 (24.6–50.0)			
Type of gastrectomy		0.128		
Total (*n* = 21, 29.2)	33.3 (15.6–51.0)			
Subtotal (*n* = 51, 70.8)	56.3 (39.8–72.7)			
Pathological findings				
T-status (AJCC 8th)		0.025		0.247
T1 (*n* = 14, 19.4)	67.1 (48.3–85.8)			
T2 (*n* = 5, 6.9)	78.3 (28.2–128.4)			
T3 (*n* = 35, 48.6)	34.2 (17.6–50.9)			
T4 (*n* = 18, 25.0)	48.4 (20.5–76.3)			
N-status (AJCC 8th)		< 0.001		0.387
N0 (*n* = 23, 31.9)	69.4 (44.9–93.8)			
N1 (*n* = 4, 5.6)	65.6 (16.7–114.5)			
N2 (*n* = 12, 16.7)	56.2 (22.8–89.6)			
N3 (*n* = 33, 45.8)	19.8 (12.9–26.6)			
M-status (AJCC 8th)		< 0.001		0.002
M0 (*n* = 59, 81.9)	58.1 (43.0–73.3)		1.000 (Reference)	
M1 (*n* = 13, 18.1)	11.0 (6.7–15.4)		3.582 (1.608–7.979)	
Lymphovascular invasion		0.001		0.085
No (*n* = 26, 36.1)	81.1 (56.6–105.6)			
Yes (*n* = 46, 63.9)	32.6 (19.8–45.5)			
Perineural invasion		< 0.001		0.006
No (*n* = 25, 34.7)	86.3 (62.2–110.3)		1.000 (Reference)	
Yes (*n* = 47, 65.3)	29.7 (17.8–41.7)		4.506 (1.541–13.177)	
Cell differentiation		0.010		
Well (*n* = 10,13.9)	108.8 (74.5–143.1)			0.229
Moderate (*n* = 26, 36.1)	32.8 (18.7–46.8)			
Poor (*n* = 36, 50.0)	43.1 (25.7–60.6)			
Maximal tumor diameter (cm)		0.038		0.343
≤5 (*n* = 40, 55.6)	59.4 (41.9–76.9)			
>5 (*n* = 32, 44.4)	36.4 (17.3–55.5)			
IHC pixelwise H-score				
TFAM IHC pixelwise H-score		0.004		0.021
Low (≤ 43.0)(*n* = 29, 40.3)	30.5 (14.2–46.8)		2.332 (1.136–4.787)	
High (> 43.0)(*n* = 43, 59.7)	63.2 (45.3–81.2)		1.000 (Reference)	
HER2 IHC pixelwise H-score		0.233		
Low (≤ 2.4)(*n* = 36, 50.0)	42.8 (25.8–59.8)			
High (> 2.4)(*n* = 36,50.0)	59.1 (38.9–79.4)			
p53 IHC pixelwise H-score		0.668		
Low (≤ 14.8)(*n* = 36, 50.0)	50.9 (33.5–68.4)			
High (> 14.8)(*n* = 36, 50.0)	51.9 (31.6–72.2)			

AJCC, American Joint Committee on Cancer; HRs, hazard ratios; GAC = gastric adenocarcinoma; IHC, immunohistochemical; TFAM, mitochondrial transcription factor A

Because HER2 and p53 IHC pixelwise H-scores were not significantly associated with mortality hazard in the continuous Cox regression models, their median values were used to dichotomize patients. The median HER2 H-score was 2.4, which was used to define low (≤ 2.4) and high (> 2.4) HER2 expression groups (*n* = 36 each). There was no significant difference between low and high HER2 groups in hazard of death (Low, HR = 1.399, 95% CI = 0.804–2.436; High, HR = 1.000, as reference, *p* = 0.235, Cox regression, uni-variate, Table 4) or overall survival (Low, mean = 42.8, 95%CI = 25.8–59.8, months; High, mean = 59.1, 95%CI = 38.9–79.4, months, *p* = 0.233, Log-rank)(Supplemental Fig. 2b; Table 5).

Similarly, the median p53 H-score was 14.8, used to define low (≤ 14.8) and high (> 14.8) p53 expression groups (*n* = 36 each). No significant differences were observed between these groups in hazard of death (Low, HR = 0.886, 95%CI = 0.509–1.542; High, HR = 1.000, as reference, *p* = 0.668, Cox regression, uni-variate, Table 4) or survival (Low, mean = 50.9, 95%CI = 33.5–68.4, months; High, mean = 51.9, 95%CI = 31.6–72.2, months, *p* = 0.668, Log-rank) (Supplemental Fig. 2c; Table 5).

### Prognostic and independent variables in Cohort B GAC patients (n = 72)

The potential prognostic variables affecting survival are summarized in Table 5. Through the comparing of Kaplan–Meier survival curves, it revealed that advanced T status (*p* = 0.025, Log-rank), advanced N status (*p* < 0.001, Log-rank), M1 status (*p* < 0.001, Log-rank), lymphovascular invasion (*p* = 0.001, Log-rank), perineural invasion (*p* < 0.001, Log-rank), moderate/poor cell differentiation (*p* = 0.010, Log-rank), a maximal tumor diameter > 5 cm (*p* = 0.038, Log-rank), and a low TFAM IHC pixelwise H-score (*p* = 0.004, Log-rank) were all associated with shorter survival (Table 5, left side).

**Fig. 4:**
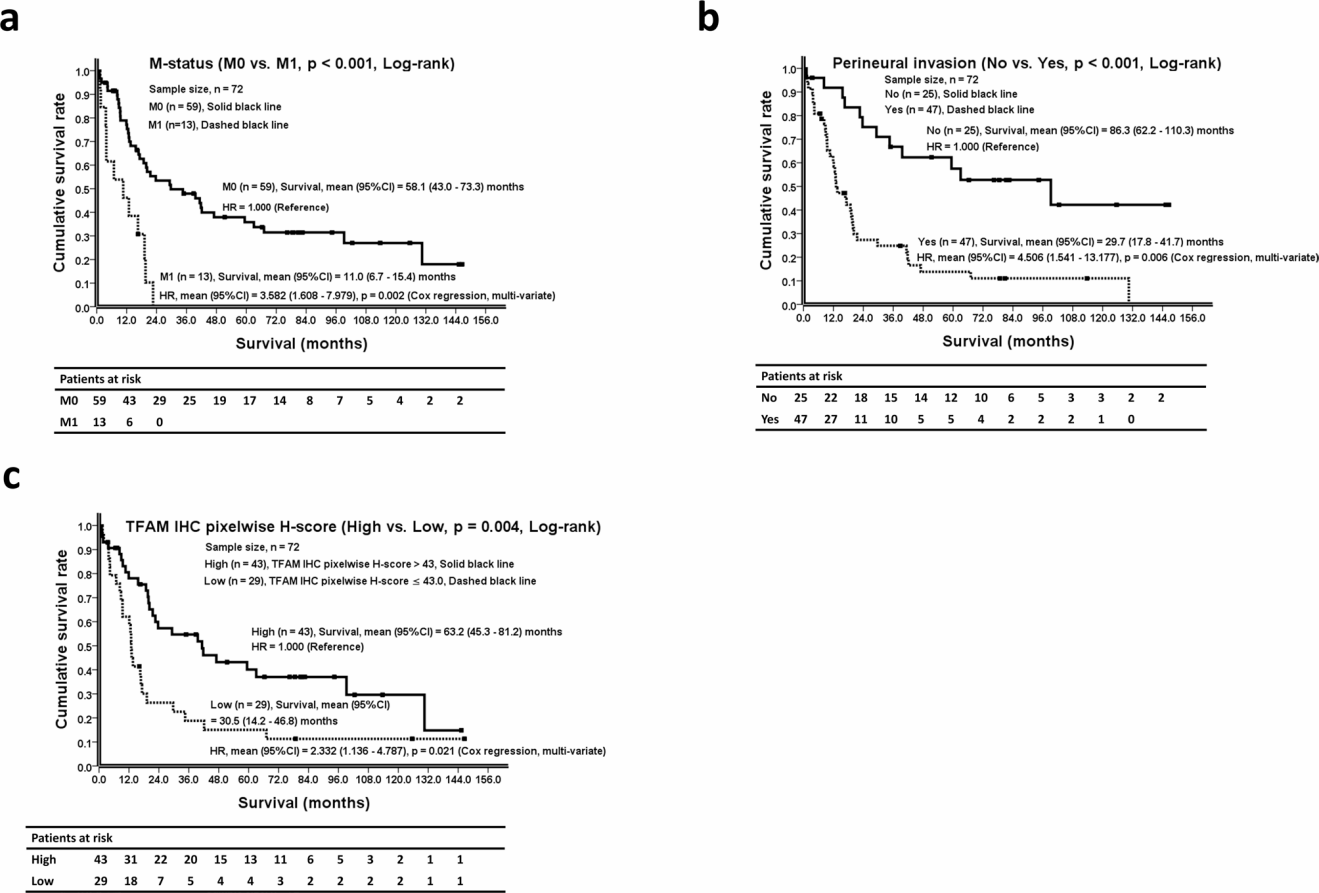
Using the Log-rank test and a multi-variate Cox regression model with the enter method, three independent predictors to distinguish survival differences and mortality risks were identified in Cohort B GAC patients (*n* = 72). (a) Kaplan–Meier survival curves demonstrate that M0 GAC patients (*n* = 59; solid black line) had a significantly better overall survival (mean = 58.1, 95%CI = 43.0–73.3, months) than did M1 patients (*n* = 13; dashed black line; mean = 11.0, 95%CI = 6.7–15.4, months, *p* < 0.001, Log-rank test). Using the M0 group as the reference (HR = 1.000), the HR for M1 patients was obviously elevated to 3.582 (95%CI = 1.608–7.979, *p* =  0.002, multi-variate Cox regression). The number of patients at risk is shown below at 12-month intervals. (b) Kaplan–Meier survival curves demonstrate that GAC patients without pleural invasion (*n* =  25 , solid black line) had a significantly better overall survival (mean = 86.3, 95%CI = 62.2–110.3, months) than did GAC patients with pleural invasion (*n* = 47, dashed black line, mean = 29.7, 95%CI = 17.8–41.7, months, *p* < 0.001, Log-rank test). Using the GAC patients without pleural invasion as the reference (HR = 1.000), the HR for GAC patients with pleural invasion was obviously elevated to 4.506 (95%CI = 1.541–13.177, *p* = 0.006, multi-variate Cox regression). The number of patients at risk is shown below at 12-month intervals. (c) Kaplan–Meier survival curves demonstrate that GAC patients with high TFAM IHC pixelwise H-score (> 43.0, *n* = 43, solid black line) had a significantly better overall survival (mean = 63.2, 95%CI = 45.3–81.2, months) than did GAC patients with low TFAM IHC pixelwise H-score (≤ 43.0, *n* = 29, dashed black line, mean = 30.5, 95%CI = 14.2–46.8, months, *p* = 0.004, Log-rank test). Using the GAC patients with high TFAM IHC pixelwise H-score (≤ 43.0) as the reference (HR = 1.000), the HR for GAC patients with low TFAM IHC pixelwise H-score (> 43.0) was obviously increased to 2.332 (95%CI = 1.136–4.787, *p* = 0.021, multi-variate Cox regression). The number of patients at risk is shown below at 12-month intervals. GAC, gastric adenocarcinoma; TFAM, mitochondrial transcription factor A; IHC, immunohistochemical; HR, hazard ratio

Variables with a *p*-value ≤ 0.1 (Log-rank test) were included in a multi-variate Cox regression model using the enter method. M1 status (HR = 3.582, 95%CI = 1.608–7.979, HR = 1.000 for M0 as reference, *p* = 0.002, Cox regression, multi-variate), perineural invasion (HR = 4.506, 95%CI = 1.541–13.177, HR = 1.000 for without perineural invasion, *p* = 0.006, Cox regression, multi-variate), and a low TFAM IHC pixelwise H-score (HR = 2.332, 95%CI = 1.136–4.787, HR = 1.000 for high TFAM IHC pixelwise H-score, *p* = 0.021, Cox regression, multi-variate) were identified as independent predictors with increased mortality risk (Table 5, right side). Along with the Kaplan-Meier survival curves, they are illustrated in Fig. 4 (a, b, & c).

### Impact of HER2 and p53 IHC pixelwise H-scores on survival HRs in Cohort B GAC patients based on independent variables (n = 72)

Although HER2 and p53 IHC pixelwise H-scores did not influence survival hazards among Cohort B GAC patients, we sought to explore their impact within each category of the three independent variables.

The results are presented in Table 6. Among the 13 M1 GAC patients, a higher p53 IHC pixelwise H-score was associated with an increased mortality HR of 1.029 (95%CI = 1.004–1.056, *p* = 0.025, Cox regression, uni-variate). In the high TFAM group, a higher HER2 IHC pixelwise H-score was associated with a higher mortality HR of 1.010 (95%CI = 1.002–1.019, *p* = 0.020, Cox regression, uni-variate).

**Table 6:** Impact of HER2 and p53 IHC pixelwise H-scores on survival HRs in Cohort B GAC patients based on independent variables

Independent variables (case number, %)	Cox regression model	
	HRs (95%CI)	*p*-value (uni-variate)
M-status		
M0 (*n* = 59, 81.9)		
HER2 IHC pixelwise H-score	1.004 (0.997–1.010)	0.297
p53 IHC pixelwise H-score	0.998 (0.989–1.007)	0.652
M1 (*n* = 13, 18.1)		
HER2 IHC pixelwise H-score	1.005 (0.990–1.020)	0.496
p53 IHC pixelwise H-score	1.029 (1.004–1.056)	0.025
Perineural invasion		
No (*n* = 25, 34.7)		
HER2 IHC pixelwise H-score	0.999 (0.954–1.046)	0.965
p53 IHC pixelwise H-score	0.996 (0.981–1.012)	0.612
Yes (*n* = 47, 65.3)		
HER2 IHC pixelwise H-score	1.001 (0.995–1.007)	0.708
p53 IHC pixelwise H-score	1.001 (0.992–1.011)	0.790
TFAM IHC pixelwise H-score		
Low (≤ 43.0) (*n* = 29, 40.3)		
HER2 IHC pixelwise H-score	0.998 (0.989–1.007)	0.697
p53 IHC pixelwise H-score	0.991 (0.977–1.007)	0.266
High (> 43.0) (*n* = 43, 59.7)		
HER2 IHC pixelwise H-score	1.010 (1.002–1.019)	0.020
p53 IHC pixelwise H-score	1.005 (0.996–1.014)	0.256

HER2, human epidermal growth factor receptor 2; HRs, hazard ratios; TFAM, mitochondrial transcription factor A; IHC, immunohistochemical

## Discussion

**Fig. 5:**
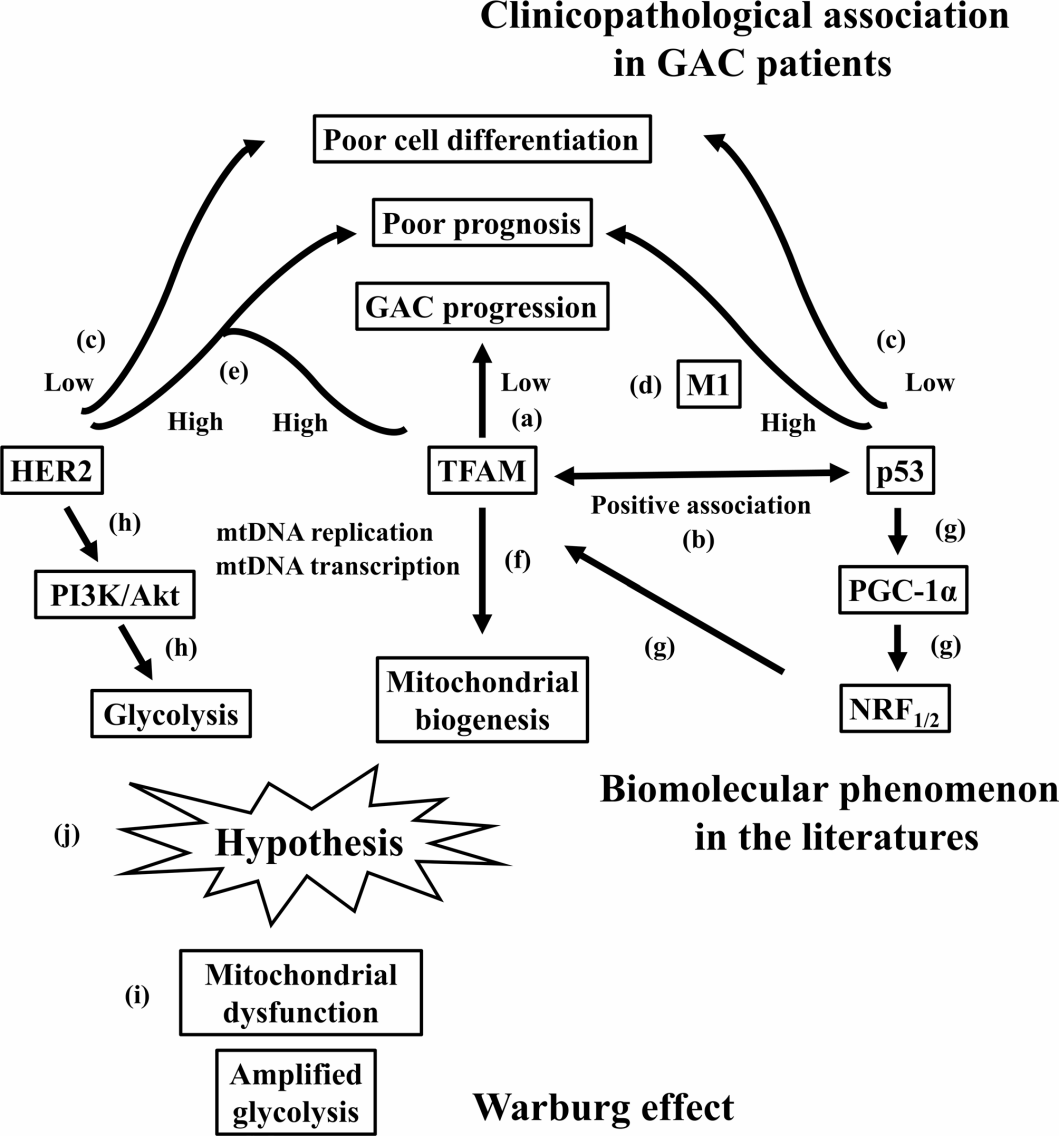
Clinicopathological observation in GAC patients, biomolecular phenomenon in the literatures and the cautious hypothesis linking to Warburg effect in GAC. Clinical observations from our cohort: (a) lower TFAM IHC pixelwise H-scores were associated with disease progression, including advanced T/N stage, lymphovascular invasion, perineural invasion, larger tumor diameter, along with poor cell differentiation and worse survival; (b) TFAM and p53 IHC pixelwise H-scores showed a positive correlation (best fit: cubic); (c) lower HER2 and p53 IHC pixelwise H-scores were also associated with poor cell differentiation; (d) among M1 patients, higher p53 IHC pixelwise H-scores were associated with increased HR; (e) within the high-TFAM subgroup, higher HER2 IHC pixelwise H-scores were associated with increased HR. Literature-derived mechanisms: (f) TFAM regulates mtDNA replication and transcription; (g) p53 can promote mitochondrial biogenesis via the PGC-1α → NRF_1/2_ → TFAM axis; (h) HER2 activates PI3K/Akt signaling to enhance glycolysis. Warburg effect and cautious hypothesis: (i) the Warburg phenotype features increased glycolysis with relative mitochondrial dysfunction; (j) we hypothesize that reduced TFAM may mark or contribute to Warburg-related progression in GAC, potentially modulated by p53 and HER2; this requires prospective validation. GAC, gastric adenocarcinoma; IHC, immunohistochemical; TFAM, mitochondrial transcription factor A; mtDNA, mitochondrial DNA; PGC-1α, peroxisome proliferator-activated receptor-γ coactivator-1α; NRF_1/2_, nuclear respiratory factors 1/2; PI3K, phosphoinositide 3-kinase; HR, hazard ratio

We had the following key findings: (1) M1 status, perineural invasion, and low TFAM IHC pixelwise H-score were independent predictors of shorter survivals with higher HRs; (2) Lower TFAM IHC pixelwise H-scores were associated with advanced T-/N-status, lymphovascular invasion, perineural invasion and poor cell differentiation, as well as with larger tumor diameter in a logarithmic pattern; (3) TFAM and p53 IHC pixelwise H-scores were positively correlated in a cubic pattern; (4) Lower IHC pixelwise H-scores of TFAM, HER2, and p53 were all associated to poor cell differentiation; (5) Higher p53 IHC pixelwise H-scores increased HRs in M1 GAC patients, while higher HER2 IHC pixelwise H-scores increased HRs in GAC patients with high TFAM IHC pixelwise H-scores. Of note, in this study, the expression levels of TFAM, HER2, and p53 were semiquantified using IHC pixelwise H-scores instead of the conventional 0/1+/2+/3+ (negative/low positive/positive/high positive) categorical scoring method. Although several statistical methods were applied in this study, these results are exploratory and were not corrected for multiple testing or comparisons. Therefore, we have summarized these clinicopathological observations in our cohort, biomolecular phenomenon in the literatures and then made a cautious hypothesis as shown in Fig. 5. Several viewpoints warranted further discussion.

In our cohort of GAC patients, aside from TFAM, neither HER2 nor p53 IHC, pixelwise H-scores demonstrated a significant prognostic effect (Tables 4 and 5; Supplemental Fig. 2, a, b & c). To provide external context, we queried the TCGA STAD cohort via the Human Protein Atlas (https://www.proteinatlas.org/).

Consistent with our institutional findings, *ERBB2* expression (https://www.proteinatlas.org/ENSG00000141736-ERBB2/cancer/stomach+cancer#STAD_TCGA) and *TP53* expression (https://www.proteinatlas.org/ENSG00000141510-TP53/cancer/stomach+cancer#STAD_TCGA) were not significantly associated with overall survival for GAC in that dataset. Nevertheless, the prognostic impact of *ERBB2* and *TP53* appeared to be cancer-type dependent. *ERBB2* expression was linked to unfavorable survival in glioma, ovarian, and pancreatic cancers but favorable survival in renal cancer (https://www.proteinatlas.org/ENSG00000141736-ERBB2/cancer), whereas *TP53* expression showed a favorable association in breast and colorectal cancers (https://www.proteinatlas.org/ENSG00000141510-TP53/cancer). In the literatures, HER2-positive GAC patients have a better prognosis than HER2-negative patients, particularly when treated with trastuzumab [28], on the contrary, HER2 expression did not affect survival in a report for Chinese patients [29]. A meta-analysis indicated overexpression of p53 detected by IHC was associated with a poor prognosis in GAC patients [30].

We demonstrated that a lower TFAM IHC expression was associated with both pathological progression (Table 2) and shorter survival (Tables 4 and 5; Supplemental Fig. 2a, Fig. 4c) in GAC patients. In contrast, the TCGA STAD dataset via Human Protein Atlas did not show a significant survival difference by TFAM expression (https://www.proteinatlas.org/ENSG00000108064-TFAM/cancer/stomach+cancer#STAD_TCGA). Based on TFAM’s critical role in mitochondrial biogenesis, our clinical observations appeared consistent with the Warburg effect in cancer progression, particularly through the lens of mitochondrial dysfunction associated with reduced TFAM expression. Previous studies have shown that TFAM knockdown can induce glucose metabolic reprogramming toward a Warburg-like phenotype, characterized by reduced oxygen consumption rate, increased extracellular acidification rate, and upregulation of glycolytic enzymes. These metabolic alterations have been demonstrated in multiple cancer cell lines, including those derived from esophageal cancer [31], renal cell carcinoma [32], colorectal cancer [33], lung cancer [24] as well as gastric cancer [34]. In contrast, their parental non-targeting control clones with higher TFAM expression retained greater oxidative phosphorylation capacity and relied less on glycolysis. Collectively, these in vitro findings suggested a TFAM-dependent divergence in metabolic phenotype. Beyond metabolic reprogramming, TFAM knockdown has also been shown to enhance migration and/or invasion in lung cancer cells [24], renal carcinoma cells [32], gastric cancer cells [34], head and neck cancer cells [35] and hepatocellular cells [36]. Clinical sample analyses further indicated that lower TFAM expression correlates with poor prognosis in patients with renal cell carcinoma, glioma, and colorectal cancer (https://www.proteinatlas.org/ENSG00000108064-TFAM/cancer). From this perspective, TFAM may function as a tumor suppressor in certain cancer types. From a translational perspective, it is plausible that TFAM expression could be incorporated into routine pathological evaluation as an adjunct biomarker. For instance, integrating TFAM status into the current AJCC staging system may help identify high-risk GAC patients beyond conventional parameters, thereby guiding oncologists toward more personalized treatment strategies.

Nevertheless, contradictory findings existed. TFAM may function as a tumor promoter in some other human cancers. In cell line studies, TFAM knockdown has been shown to reduce migration and/or invasion in esophageal cancer cells [31], colorectal cancer cells [33] and lung cancer cells [37]. Although not significant, clinical sample analyses indicated that high TFAM expression is potentially associated with poor prognosis in liver cancer and pancreatic cancer (as reported in the Human Protein Atlas, https://www.proteinatlas.org/ENSG00000108064-TFAM/cancer/liver+cancer#LIHC_validation; https://www.proteinatlas.org/ENSG00000108064-TFAM/cancer/pancreatic+cancer#PAAD_TCGA). These findings suggest that TFAM’s role in cancer progression may be context-dependent, acting as either a tumor promoter or a suppressor depending on the cancer type, cellular environment, or molecular interactions. To advance TFAM’s clinical application, multiple aspects should be critically appraised and systematically evaluated in future studies.

Taken together, differences in analyzed populations, the molecular level of assessment (DNA, RNA, or protein), and study aims might account for the above discrepancies in TFAM, HER2 and p53 studies. We acknowledge that these analyses remain correlative and that the current study is observational in nature. The lack of direct functional or mechanistic experiments limited causal inference. Future work with larger cohorts and in vitro/in vivo modulation of TFAM, p53 and HER2 would be essential to clarify their roles and interactions in GAC.

To further explore this context specificity, we conducted a more detailed analysis, which revealed that tumor diameter, representing an environmental factor, and p53 IHC pixelwise H-score, representing a biological factor, exhibited distinct associated with TFAM IHC pixelwise H-score among GAC samples. Specifically, TFAM IHC pixelwise H-score was negatively correlated with tumor diameter in a logarithmic pattern, and positively correlated with p53 IHC pixelwise H-score in a cubic pattern (Table 3; Fig. 3).

Consistent with a prior study, a negative association between tumor size and TFAM expression has been reported in lung cancer [24]. A larger tumor diameter is often indicative of progressive central tumor hypoxia, as rapidly proliferating cancer cells outgrow the development of new blood vessels [38, 39]. Hypoxia in the tumor microenvironment not only drives tumor progression but is also linked to the suppression of TFAM expression. Notably, one study demonstrated that hypoxic conditions reduced TFAM gene expression by 30% after 24 h and by 44% after 42 h in human pulmonary artery endothelial cells [40]. These findings suggest that hypoxic conditions in larger tumors may suppress TFAM expression, leading to mitochondrial dysfunction and promoting the Warburg effect in human cancers.

Regarding the cubic relationship between p53 and TFAM among GAC samples, our analysis revealed dynamic fluctuations in p53 levels, characterized by an initial decline, reaching a low valley, followed by a rebound as TFAM expression increases. Additionally, an elevated p53 expression was associated with a worsened prognostic hazards in M1 GAC patients (Table 6). These observations suggest a complex regulatory interplay between p53 and TFAM in GAC, potentially reflecting distinct functional phases that may influence tumor progression and patient outcomes. Given the diverse roles of p53 in cancer biology, understanding this p53-TFAM interaction is crucial for elucidating its role in cancer metabolism and the Warburg effect.

The primary antibody (clone DO7) used in this study detects both wild-type and mutant p53, making it challenging to distinguish wild-type p53 stabilization in response to cellular stress from the abnormal accumulation of mutant p53 due to impaired degradation mechanisms [41]. Under normal physiological conditions, wild-type p53 is maintained at low levels due to continuous proteasomal degradation mediated by MDM2, an E3 ubiquitin ligase that facilitates p53 breakdown. However, upon exposure to cellular stressors, such as DNA damage, hypoxia, or oncogene activation, the MDM2-p53 interaction is disrupted, leading to p53 stabilization and accumulation. This allows wild-type p53 to function as a transcription factor, regulating genes involved in cell cycle arrest, DNA repair, and apoptosis, thereby playing a crucial role in genomic integrity maintenance [42]. In contrast, *TP53* mutations, which are frequently observed in human cancers, result in the production of mutant p53 proteins that are resistant to degradation, leading to their abnormal accumulation. Unlike wild-type p53, mutant p53 may lose its tumor-suppressive functions and, in some cases, acquire oncogenic gain-of-function (GOF) properties, thereby promoting tumor progression, metastasis, and therapy resistance [43]. Additionally, some mutant p53 proteins can exert a dominant-negative effect, interfering with wild-type p53 activity and further exacerbating oncogenesis [44]. Concerning the potential role of p53 in TFAM-related mitochondrial function, studies have demonstrated that p53 can enhance mitochondrial biogenesis through its interaction with TFAM, highlighting its role in mitochondrial homeostasis [45]. Conversely, mutant p53 has been shown to interact with the TFAM gene to maintain mtDNA transcription, even under stress conditions such as exposure to doxorubicin [46].

As a result, the interpretation of our p53 IHC findings should be made with caution, given that the DO7 antibody used in this study cannot differentiate between wild-type and mutant forms of p53. Consequently, the observed p53 immunopositivity cannot be directly interpreted as indicating *TP53* mutation. We acknowledge this as a key limitation of our study. To address this, *TP53* mutational testing using DNA extracted from formalin-fixed, paraffin-embedded tissue blocks represents a technically feasible and commonly used approach in molecular pathology [47]. The integration of p53 IHC with molecular techniques, such as Sanger sequencing or next-generation sequencing (NGS) panels [][47], may provide a more accurate and comprehensive characterization of *TP53* status in GAC. Future studies incorporating such molecular profiling are warranted to clarify the underlying nature of p53 expression and to further elucidate the p53/PGC-1α/TFAM signaling axis. These efforts will be essential to validate our hypothesis and to better understand the implications of p53 dysfunction in relation to the Warburg effect, mitochondrial dysregulation, tumor metabolism, and the development of targeted therapies.

In our study, high TFAM expression emerged as an independent indicator of favorable prognosis in GAC patients. However, this prognostic benefit appeared to be attenuated in cases with elevated HER2 expression (Tables 5 and 6). This suggests that HER2 may represent another molecular factor contributing to the context-dependent role of TFAM in cancer progression. Previous research has demonstrated that the HER2/Akt signaling pathway plays a crucial role in driving the Warburg effect, where Akt activation promotes glucose uptake and glycolysis, resulting in increased lactate production even under normoxic conditions or in the presence of non-dysfunctional mitochondria [19]. This interplay suggests that simultaneous oxidative phosphorylation (reflected by TFAM) and glycolysis (driven by HER2/Akt) might actually exacerbate GAC aggressiveness. A similar concept was observed in a lung cancer study, which reported that coordinated increased in oxidative metabolism, indicated by markers such as PDH, combined with enhanced glycolysis via LDH signaling are associated with worse clinical outcomes [48]. Therefore, several scientists proposed that the switch toward glycolysis isn’t necessarily a compensatory mechanism for defective mitochondrial respiration, even when mitochondrial oxidative phosphorylation remains largely normal [49].

Interestingly, our results demonstrated that lower expressions of TFAM, p53, and HER2 were each associated with poor cancer differentiation (Table 2). A similar trend was reported by Zang et al., who analyzed 249 gastric cancer patients and found that p53 and HER2 expression levels were correlated with tumor grade [50]. However, no studies have specifically reported an association between lower TFAM expression and poor cancer cell differentiation. Further research is necessary to elucidate the underlying mechanisms.

Perineural invasion, referring cancer cells that invade through perineural spaces surrounding nerves, has been identified as an independent and poor prognostic factor in the current study (Table 5). An important meta-analysis about 30,590 gastric cancer patients undergoing curative gastrectomy also revealed that perineural invasion was significantly correlated with decreased overall survival and disease-free survival, independent of other variables such as lymph node status, tumor size, and depth of invasion [51]. The adverse prognostic impact of perineural invasion has also been reported in other malignancies, including colorectal cancer [52] and head and neck cancer [53]. Although perineural invasion is well recognized as a negative prognostic indicator in GAC, its relationship with TFAM, p53, and HER2 expression remained unclear and it needed further investigation.

Traditionally, most research has emphasized the primary role of mitochondria as the cellular powerhouse responsible for energy production. However, emerging evidence increasingly highlights the metabolic plasticity and functional versatility of mitochondria, positioning them as multifunctional hubs in nutritional balance through glucose metabolic reprogramming [8] or key regulators of immune responses [54]. From a clinical perspective, recent investigations have underscored the prognostic value of immune- and nutrition-related biomarkers in cancer. Examples include the modified Glasgow Prognostic Score and pan-immune-inflammation value or risk score, which have demonstrated predictive relevance in breast and colorectal cancers [55, 56, 57]. In parallel, emerging immune-metabolic regulators such as IL27RA and TMEM71 have been identified through single-cell and integrative transcriptomic analyses as potential prognostic and therapeutic indicators in breast cancer [58, 59]. Given its pivotal role in mitochondrial homeostasis, these findings may provide conceptual support for the prognostic and translational potential of TFAM in GAC.

In this study, we employed the IHC pixelwise H-score to semiquantify protein expression levels in IHC. Compared to the conventional categorical IHC scoring system (0, 1^+^, 2^+^, 3^+^), the IHC pixelwise H-score provides several advantages. First, it offers a semiquantitative and continuous assessment of protein expression by integrating both staining intensity and the proportion of stained cells, thereby increasing sensitivity to subtle expression differences. Second, it reduces interobserver variability and subjectivity by utilizing digital image analysis, enhancing reproducibility. Third, the continuous nature of the IHC pixelwise H-score allows for more robust statistical analyses, including correlation and regression models, which are often limited with ordinal data [23]. To validate these advantages, we used HER2 expression as an internal benchmark, given its broader dynamic range, reflected by a substantially higher mean compared to median IHC pixelwise H-score, relative to TFAM and p53 (Table 1). HER2 IHC results, categorized by traditional scoring (0, 1+, 2+, 3+), corresponded to progressively increasing mean IHC pixelwise H-scores: 1.8, 3.9, 22.3, and 121.9, respectively (*p* < 0.001, Kruskal-Wallis *H* test). A strong positive correlation between the categorical scores and IHC pixelwise H-scores was observed (Spearman’s rho = 0.779, *p* < 0.001), supporting the consistency and reliability of this semiquantitative method (Supplemental Fig. 3). Furthermore, in the uni-variate Cox regression analysis of HER2 within the high-TFAM subgroup, a higher IHC pixelwise H-score was significantly associated with increased HR of death (*p* = 0.020; HR = 1.010, 95% CI = 1.002–1.019, Table 6). In contrast, the conventional categorical HER2 score (0/1^+^/2^+^/3^+^) showed no significant association with survival HR (*p* = 0.404, data not shown). Collectively, the IHC pixelwise H-score approach enhances the precision, objectivity, and statistical power of IHC biomarker evaluation, offering clear advantages over the conventional categorical (0–3^+^) scoring system. This semiquantitative method aligns with the growing trend of AI-assisted digital pathology by transforming IHC staining results into continuous, quantifiable data. Such digitized output enables seamless integration with image analysis algorithms and machine learning frameworks, thereby improving diagnostic consistency, supporting high-throughput biomarker profiling, and paving the way for automated, reproducible assessments in precision oncology [60].

## Conclusions

Our findings demonstrate that low TFAM expression is associated with more aggressive clinicopathological features and reduced overall survival in patients with GAC. Although these observations imply TFAM’s potential as a prognostic biomarker, the retrospective and observational design precludes causal inferences or firm conclusions about underlying metabolic mechanisms. The correlations we observed between TFAM, p53, and HER2 expression are hypothesis-generating and require prospective validation in larger, independent cohorts. Further functional studies will be essential to confirm TFAM’s role in metabolic reprogramming and to establish its clinical utility as a biomarker in GAC.

## Supplementary Information

Below is the link to the electronic supplementary material.

Supplementary file 1
Supplemental Fig. 1. IHC staining controls for TFAM, HER2, and p53 in Cohort A GAC patients. Left panels: Tumor regions with positive DAB staining for (a) TFAM, (b) HER2, and (c) p53. Right panels: Internal negative controls from non-neoplastic tissue (adipose, muscle, fibrous stroma, or vascular adventitia) processed in parallel, showing only hematoxylin (blue) counterstain and no obvious DAB signal. DAB, 3,3′-diaminobenzidine; IHC, immunohistochemical; TFAM, mitochondrial transcription factor A; HER2, human epidermal growth factor receptor 2.

Supplementary file 2
Supplemental Fig. 2. Prognostic impact of TFAM, HER2, and p53 IHC pixelwise H-scores in Cohort B GAC patients (n = 72). (a) TFAM. Patients were dichotomized by the TFAM IHC pixelwise H-score cutoff of 43.0 into high (n = 43) and low (n = 29) expression groups. Kaplan–Meier curves show that low-TFAM patients had shorter overall survival (mean = 30.5, 95% CI = 14.2–46.8, months) compared with high-TFAM patients (mean = 63.2, 95%CI = 45.3–81.2, months)(*p* = 0.004, Log-rank test). In uni-variate Cox regression, using the high-TFAM group as reference (HR = 1.000), the low-TFAM group had an elevated HR of 2.223 (95% CI = 1.272–3.884, *p* = 0.005). Patients at risk are shown below at 12-month intervals. (b) HER2. Patients were dichotomized by the median HER2 H-score of 2.4 into high (n = 36) and low (n = 36) groups. Low-HER2 patients had a mean survival of 42.8 months (95% CI = 25.8–59.8) versus 59.1 months (95%CI = 38.9–79.4) in the high-HER2 group (*p* = 0.233, Log-rank test). In uni-variate Cox regression, using the high-HER2 group as reference (HR = 1.000), the low-HER2 group had HR = 1.399 (95%CI = 0.804–2.436, *p* = 0.235). Patients at risk are shown below. (c) p53. Patients were dichotomized by the median p53 H-score of 14.8 into high (n = 36) and low (n = 36) groups. Low-p53 patients had a mean survival of 50.9 months (95% CI = 33.5–68.4) versus 51.9 months (95%CI = 31.6–72.2) in the high-p53 group (*p* = 0.668, Log-rank test). In uni-variate Cox regression, using the high-p53 group as reference (HR = 1.000), the low-p53 group had HR = 0.886 (95%CI = 0.509–1.542, *p*= 0.668). Patients at risk are shown below. GAC, gastric adenocarcinoma; IHC, immunohistochemical; HR, hazard ratio; CI, confidence interval.

Supplementary file 3
Supplemental Fig. 3. Correlation between conventional HER2 IHC category and pixelwise H-scores in GAC. Bar charts illustrate the distribution of HER2 pixelwise H-scores across conventional IHC scoring categories (0, 1^+^, 2^+^, 3^+^) in GAC samples. Mean pixelwise H-scores increased stepwise with higher IHC categories: 1.8 for 0 (n = 20), 3.9 for 1^+^ (n = 30), 22.3 for 2^+^ (n = 19), and 121.9 for 3^+^ (n = 8)(*p* < 0.001, Kruskal–Wallis *H* test). A strong positive correlation was observed between the conventional categorical scores and pixelwise H-scores (Spearman’s rho = 0.779, *p* < 0.001). HER2, human epidermal growth factor receptor 2; GAC, gastric adenocarcinoma; IHC, immunohistochemical.

## Data Availability

The datasets used and analyzed during the current study are available from the corresponding authors on reasonable request.

## Abbreviations

AJCC: American Joint Committee on Cancer
ANOVA: Analysis of variance
AUC: Area under the curve
Akt: Protein kinase B
CI: Confidence interval
DAB: 3,3′-Diaminobenzidine
GAC: Gastric adenocarcinoma
HER2: Human epidermal growth factor receptor 2
HR: Hazard ratio
IHC: Immunohistochemical
IHC pixelwise H-score: A digital image analysis-based metric to semiquantify protein/biomarker expression level from immunohistochemical (IHC) images
mtDNA: Mitochondrial DNA
nDNA: Nuclear DNA
NRF1/2: Nuclear respiratory factors 1 and 2
PGC-1α: Peroxisome proliferator-activated receptor gamma coactivator 1-alpha
PI3K: Phosphoinositide 3-kinase
ROC: Receiver operating characteristic
STD: Standard deviation
TFAM: Mitochondrial transcription factor A
WSI: Whole slide image
