# Supplementary figures and images for: Semiquantitative immunohistochemical (IHC) pixelwise H-score of mitochondrial transcription factor A (TFAM) in gastric adenocarcinoma (GAC): clinicopathological significance and association with p53 and HER2
Source: World J Surg Oncol. 2025 Dec 15;23:452. doi: 10.1186/s12957-025-03998-6 (PMC12707001; doi:10.1186/s12957-025-03998-6)

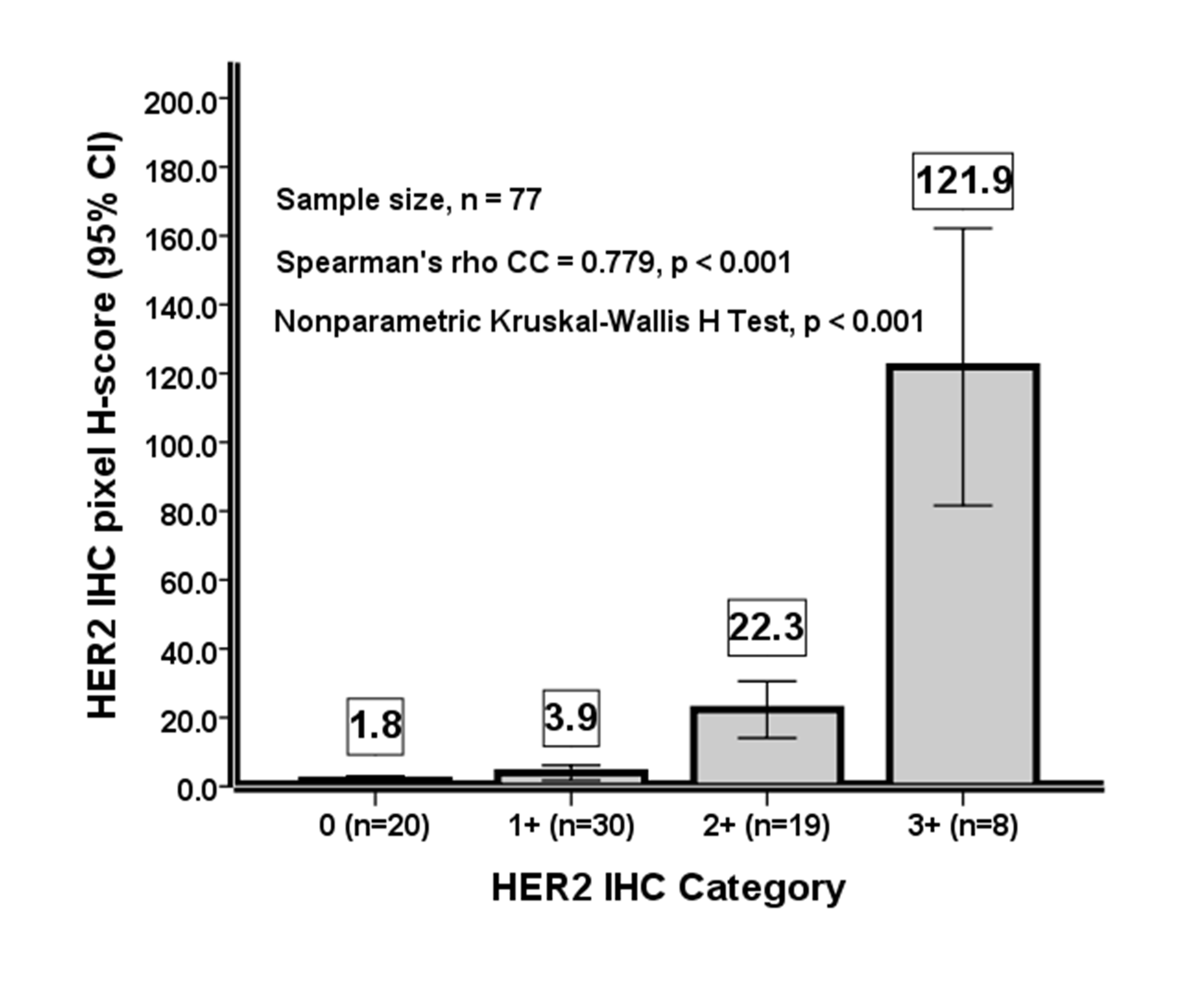

Supplement: Supplementary file 3 — Supplemental Fig. 3. Correlation between conventional HER2 IHC category and pixelwise H-scores in GAC. Bar charts illustrate the distribution of HER2 pixelwise H-scores across conventional IHC scoring categories (0, 1+, 2+, 3+) in GAC samples. Mean pixelwise H-scores increased stepwise with higher IHC categories: 1.8 for 0 (n = 20), 3.9 for 1+ (n = 30), 22.3 for 2+ (n = 19), and 121.9 for 3+ (n = 8)(p < 0.001, Kruskal–Wallis H test). A strong positive correlation was observed between the conventional categorical scores and pixelwise H-scores (Spearman’s rho = 0.779, p < 0.001). HER2, human epidermal growth factor receptor 2; GAC, gastric adenocarcinoma; IHC, immunohistochemical. [file 12957_2025_3998_MOESM3_ESM.tif]
